# Anti-TNF therapy for ulcerative colitis in Brazil: a comparative real-world national retrospective multicentric study from the Brazilian study group of IBD (GEDIIB)

**DOI:** 10.1186/s12876-022-02341-7

**Published:** 2022-05-29

**Authors:** Ligia Yukie Sassaki, Daniela Oliveira Magro, Rogerio Saad-Hossne, Julio Pinheiro Baima, Cristina Flores, Lucianna Motta Correia, Lívia Medeiros Soares Celani, Maria De Lourdes De Abreu Ferrari, Patricia Zacharias, Marley Ribeiro Feitosa, Carlos Henrique Marques Dos Santos, Manoel Alvaro De Freitas Lins Neto, Abel Botelho Quaresma, Sergio Figueiredo De Lima Junior, Graciana Bandeira Salgado De Vasconcelos, Ornella Sari Cassol, Arlene Dos Santos Pinto, Gustavo Kurachi, Francisco de Assis Goncalves Filho, Rodrigo Galhardi Gasparini, Thaísa Kowalski Furlan, Wilson Roberto Catapani, Cláudio Saddy Rodrigues Coy, Vivian De Souza Menegassi, Marilia Majeski Colombo, Renata de Sá Brito Fróes, Fabio Vieira Teixeira, Antonio Carlos Moraes, Genoile Oliveira Santana, José Miguel Luz Parente, Eduardo Garcia Vilela, Natália Sousa Freitas Queiroz, Paulo Gustavo Kotze, GEDIIB (Brazilian Study Group of IBD)

**Affiliations:** 1 Department of Internal Medicine, Medical School, São Paulo State University (UNESP), Botucatu, Brazil; 2 Colorectal Surgery Unit, University of Campinas UNICAMP, Campinas, Brazil; 3 Department of Surgery, Medical School, São Paulo State University Unesp, Botucatu, Brazil; 4 Hospital de Clínicas de Porto Alegre, Porto Alegre, Brazil; 5 Onofre Lopes Universitary Hospital, Federal University of Rio Grande Do Norte, Natal, Brazil; 6 Medical School of the Federal University of the Minas Gerais, Belo Horizonte, Brazil; 7 IBD Outpatient Clinics- Colorectal Surgery Unit, Catholic University or Paraná PUCPR, Curitiba, Brazil; 8 Department of Surgery and Anatomy, Ribeirao Preto Medical School, University of Sao Paulo, Ribeirao Preto, Brazil; 9 Surgery Department, Universidade Federal de Mato Grosso Do Sul, Campo Grande, Brazil; 10 Federal University of Alagoas, Maceio, Brazil; 11 Surgery, Universidade Do Oeste de Santa Catarina UNOESC, Joaçaba, Brazil; 12 Colorectal Surgery Unit, João de Barros Barreto University Hospital, Federal University of Pará, Belém, Brazil; 13 Gastroenterologia, Fundação Universidade de Pernambuco, Recife, Brazil; 14 Hospital de Clínicas de Passo Fundo, Passo Fundo, Brazil; 15 Hospital Universitario Getulio Vargas, Manaus, Brazil; 16 Gastroenterology, Gastroclinica Cascavel, Cascavel, Brazil; 17 Department of surgery, Faculty of Medicine of São José do Rio Preto, São José do Rio Preto, SP Brazil; 18 SETE - Specialized Medical Center, Marília, São Paulo, Brazil; 19 Gastroenterology, Hospital de Clínicas da Universidade Federal do Paraná - HCUFPR, Curitiba, Brazil; 20 Gastroenterology, Faculdade de Medicina do ABC, Santo André, Brazil; 21 Hospital Universitário Professor Polydoro Ernani de São Thiago da Universidade Federal de Santa Catarina HU-UFSC, Florianópolis, Santa Catarina Brazil; 22 Gastroenterology, Hospital Doutor Dório Silva, Serra, Brazil; 23 Gastroenterology, Gastromed, Rio de Janeiro, Brazil; 24 GastroSaude Clinic, Marilia, Sao Paulo, Brazil; 25 Hospital Copa D’Or, Rio de Janeiro, Brazil; 26 Bahia State University UNEB, Salvador, Bahia Brazil; 27 Gastroenterology Division, Medical Health Center, Federal University of Piaui, Teresina, Brazil; 28 Gastroenterology, Hospital of the Federal University of Minas Gerais, Belo Horizonte, Brazil

**Keywords:** Anti-TNF therapy, Adalimumab, Infliximab, Clinical remission, Ulcerative colitis

## Abstract

**Background:**

Anti-TNF therapy represented a landmark in medical treatment of ulcerative colitis (UC). There is lack of data on the efficacy and safety of these agents in Brazilian patients. The present study aimed to analyze rates of clinical and endoscopic remission comparatively, between adalimumab (ADA) and infliximab (IFX), in Brazilian patients with UC, and evaluate factors associated with clinical and endoscopic remission after 1 year of treatment.

**Methods:**

A national retrospective multicenter study (24 centers) was performed including patients with UC treated with anti-TNF therapy. Outcomes as clinical response and remission, endoscopic remission and secondary loss of response were measured in different time points of the follow-up. Baseline predictive factors of clinical and endoscopic remission at week 52 were evaluated using logistic regression model. Indirect comparisons among groups (ADA and IFX) were performed using Student's t, Pearson χ^2^ or Fisher's exact test when appropriated, and Kaplan Meier analysis.

**Results:**

Overall, 393 patients were included (ADA, n = 111; IFX, n = 282). The mean age was 41.86 ± 13.60 years, 61.58% were female, most patients had extensive colitis (62.40%) and 19.39% had previous exposure to a biological agent. Overall, clinical remission rate was 66.78%, 71.62% and 82.82% at weeks 8, 26 and 52, respectively. Remission rates were higher in the IFX group at weeks 26 (75.12% vs. 62.65%, *p* < 0.0001) and 52 (65.24% vs. 51.35%, *p* < 0.0001) when compared to ADA. According to Kaplan–Meier survival curve loss of response was less frequent in the Infliximab compared to Adalimumab group (*p* = 0.001). Overall, endoscopic remission was observed in 50% of patients at week 26 and in 65.98% at week 52, with no difference between the groups (*p* = 0.114). Colectomy was performed in 23 patients (5.99%). Age, non-prior exposure to biological therapy, use of IFX and endoscopic remission at week 26 were associated with clinical remission after 52 weeks. Variables associated with endoscopic remission were non-prior exposure to biological therapy, and clinical and endoscopic remission at week 26.

**Conclusions:**

IFX was associated with higher rates of clinical remission after 1 year in comparison to ADA. Non-prior exposure to biological therapy and early response to anti-TNF treatment were associated with higher rates of clinical and endoscopic remission.

## Background

Ulcerative colitis (UC) is a chronic and progressive immune-mediated inflammatory bowel disease (IBD) which can affect a variable extension of the large bowel and predominantly impacts individuals in the third and fourth decades of life [1]. The course of the disease is characterized by relapsing and remitting mucosal inflammation and up to 15% of patients may develop severe disease at diagnosis [2]. The aim of treatment is to induce and maintain clinical and endoscopic remission, but the adoption of evolving treatment targets such as histological healing has recently been considered [3]. Biological agents are indicated for patients with moderate to severely active disease, refractory to conventional treatment with aminosalicylates and immunomodulators, as well as for those who are steroid-dependent or refractory [4].

The most commonly used tumor necrosis factor (TNF) alpha inhibitors in UC are infliximab (IFX) and adalimumab (ADA). Golimumab is also approved in UC, but experience with this agent in Brazil is limited. The efficacy of IFX in inducing and maintaining remission in moderate to severe UC, in addition to reducing the need for colectomy is supported by the pivotal ACT 1 and ACT 2 studies [5]. Accordingly, data from the pivotal Phase 3 ULTRA I and II studies demonstrated that treatment with ADA was able to induce and maintain remission in patients with moderate to severe UC and was well tolerated [6].

Although pivotal studies represent an important source of data on the efficacy and safety of a new treatment, their selective designs, with multiple inclusion and exclusion criteria, prevent the generalization of findings for routine clinical practice. Therefore, real world studies are warranted. Even though several European and North American studies have demonstrated real-world experience on the efficacy and safety of anti-TNF agents in the management of UC [7–11], the phenotype of disease can differ in various ethnic groups, which may be associated to different genetic backgrounds [12]. Thus, it is crucial to have data of specific drug efficacy in diverse populations with different demographic and socioeconomic background, such as Latin America. Possible differences in comparison with North American or European populations may limit extrapolation of available data, what emphasizes the need for specific local studies.

In this scenario, there is lack of data on the efficacy and safety of anti-TNF agents in Brazilian patients in UC, as public and private access to these agents is relatively recent [13]. Therefore, the present study aimed to analyze rates of clinical and endoscopic remission comparatively, between ADA and IFX, in Brazilian UC patients, and evaluate possible factors associated with clinical and endoscopic remission after 1 year of treatment.

## Methods

### Study design

A national retrospective multicenter study (24 centers) was carried out initially including 424 patients with UC treated with anti-TNF therapy (ADA or IFX). No patients with golimumab were included. Data were collected electronically from patient records. Inclusion criteria were clinical, endoscopic and/or histological criteria for UC diagnosis; adult patients (age over 18 years old); use of ADA or IFX due to active UC in any phase of treatment. Patients with indeterminate colitis or Crohn's disease (CD), hospitalized patients, or those who had undergone previous colorectal surgery, or with lack of essential data were excluded. Patients were allocated into 2 groups (ADA or IFX) and a comparative study was performed.

### Included variables

We analyzed sex, age, age at diagnosis, body mass index, disease duration, active smoking, associated comorbidities, concomitant or previous extraintestinal manifestations (EIM), use of corticosteroids at the treatment initiation (as co-induction), concomitant use of azathioprine, and previous exposure to biological therapy. The extent of the disease was classified according to the Montreal classification (E1: proctitis; E2: left-sided colitis and E3: extensive colitis) [14]. Disease activity was assessed using the partial Mayo score [15] at baseline, weeks 8, 26 and 52, or at the last visit. In addition, the need for colectomy during follow-up, loss of response until week 52 of treatment, and presence of adverse events were evaluated. Biochemical data such as hematocrit (%), hemoglobin (g/dl), albumin (g/dl), C-reactive protein (mg/dl), and fecal calprotectin (μg/g) were additionally evaluated, when available.

### Definitions and outcomes

The primary outcome was the proportion of patients achieving clinical remission at weeks 8, 26 and 52. Secondary outcomes were clinical response, endoscopic remission, and rates of secondary loss of response. Clinical remission was defined as partial Mayo score ≤ 2. Clinical response was defined as a reduction on partial Mayo subscore ≥ 2 points between baseline and weeks 8, 26, and 52. Endoscopic remission was defined as endoscopic Mayo subscore ≤ 1. Secondary loss of response was defined as a need for one of the following outcomes during follow-up: colectomy, dose optimization, need for corticosteroids as rescue therapy or a switch to another biological agent.

### Statistical analysis

Data were reported using “as-observed” analysis, with the denominator being the total number of patients with available data in the pre-established time points. For quantitative variables with normal distribution, mean and standard deviation (SD) were presented, and Student's t test was used to compare two independent samples. Categorical data were presented as percentages, and Pearson χ^2^ or Fisher's exact test were used to compare two proportions (from independent samples). Univariate logistic regression was used to identify predictors on categorical outcomes, such as presence or absence of remission at week 52. Survival analysis was performed using Kaplan–Meier curves and log-rank test; the considered outcomes were loss of response and colectomy. Values of *p* < 0.05 were considered statistically significant. The statistical analyses were performed using IBM SPSS v. 22.0 (UNICOM Global, Mission Hills, United States).

### Ethical considerations

This study was approved by the Local Research Ethics Committee, Botucatu Medical School (CAAE: 13,973,519.0.1001.5411) and by all respective boards from participating centers (listed in Declarations).

## Results

### Clinical characteristics

A total of 424 patients were initially evaluated, and 31 were excluded for lack of essential data. A total of 393 patients were included in the full analysis. Baseline characteristics are described in detail in Table 1. The mean age was 41.86 ± 13.60 years, 61.58% were female and 39.13% had associated comorbidities, the most frequent being hypertension (12.79%). Regarding the type of anti-TNF agent, 111 patients received ADA and 282 received IFX. Most patients had extensive colitis (62.40%) and 19.39% were previously exposed to a biological agent. The frequency of EIMs was higher in the ADA group. Co-induction with corticosteroids and the use of azathioprine in combination therapy were more frequent in the IFX group. In the comparative analysis between treatments, a higher frequency of moderate to severe endoscopic activity was observed in the IFX group at baseline.

**Table 1:** Baseline characteristics of patients with UC treated with ADA or IFX

	Adalimumab (ADA) (n=111)	Infliximab (IFX) (n=282)	P-value
Age (y)	42.95 ±14.09	41.43 ±13.40	0.3191
Age at diagnosis (y)	34.42 ±13.72	32.68 ±12.74	0.2352
BMI (kg/m^2^)	25.23 ±4.11	25.14 ±5.17	0.8771
Female gender	75 (67.57)	167 (59.22)	0.1256
Caucasian	61 (54.95)	198 (70.21)	0.0025
Active smoking	6 (5.61)	28 (10.11)	<0.001
Presence of comorbidities	43 (38.74)	110 (39.29)	0.9204
*Disease extension*			
Pancolitis	71 (64.55)	173 (61.57)	0.8599
Left sided colitis	33 (30.00)	91 (32.38)	
Distal colitis	6 (5.45)	17 (6.05)	
Extraintestinal manifestations (EIM)	46 (42.59)	91 (32.50)	<0.001
Rheumatological	31 (28.70)	60 (21.43)	0.002
Dermatological	7 (6.48)	14 (5.00)	0.127
Hepatic	7 (6.48)	9 (3.21)	0.617
Ocular	0	3 (1.07)	0.5633
Thromboembolic events	3 (2.78)	8 (2.86)	0.132
Corticosteroids at baseline	68 (61.82)	209 (74.64)	<0.001
Concomitant azathioprine	65 (58.56)	234 (82.98)	<0.0001
Time between diagnosis and onset of anti-TNF (y)	6.33 ±5.65	5.73 ±5.39	0.240
Previous use of biological therapy	36 (32.43)	40 (14.23)	0.643
*Clinical disease activity*			
Remission	2 (1.94)	6 (2.21)	0.2853
Mild	18 (17.48)	28 (10.33)	
Moderate	65 (63.11)	179 (66.05)	
Severe	18 (17.48)	58 (21.40)	
*Mayo endoscopic subscore*			
0 - remission	3 (2.91)	-	0.002
1 - mild	7 (6.79)	8 (2.94)	
2 - moderate	27 (26.20)	109 (40.07)	
3 - severe	66 (64.08)	155 (56.98)	

Data presented as mean ± SD and n (%). *BMI* Body mass index. *Calculated by Student *t* test or Pearson, χ^2^ or Fisher´s exact test

Efficacy data from the whole sample is illustrated in detail in Table 2. Overall clinical remission rates were 66.78% at week 8, 71.62% at week 26 and 82.82% at week 52, respectively. Overall clinical response rates were 61.25% at week 8, 83.85% at week 26 and 87.46% at week 52, respectively. Endoscopic remission was observed in 50% of patients at week 26 and 65.98% at week 52. Additionally, there was a decrease in the Mayo score throughout the study period and an improvement in biochemical parameters.

**Table 2:** Evaluation of Mayo score, clinical response, clinical remission, endoscopic activity, and biochemical tests throughout treatment with anti-TNF therapy

	Baseline (n=374)	Week 8 (n=352)	Week 26 (n=296)	Week 52 (n=291)
Partial Mayo score	5.75 ± 2.33	3.36 ± 2.37*	2.46 ± 2.26*	1.89 ± 2.25*
Mayo endoscopic subscore	2.53 ± 0.61	–	1.43 ± 0.97*	1.08 ± 1.10*
Full Mayo score	8.37 ± 2.48	–	3.9 ± 2.76*	2.88 ± 2.86*
*Clinical disease activity*				
Remission	8 (2.14)	90 (25.57)	115 (38.85)	162 (55.67)
Mild	46 (12.30)	145 (41.19)	97 (32.77)	79 (27.15)
Moderate	244 (65.24)	97 (27.56)	80 (27.03)	43 (14.78)
Severe	76 (20.32)	20 (5.68)	4 (1.35)	7 (2.41)
Clinical response	–	215 (61.25)	244 (83.85)	244 (87.46)
Clinical remission	54 (14.44)	235 (66.78) *	212 (71.62)	241 (82.82)
*Mayo endoscopic subscore*				
0 - remission	3 (0.80)	–	47 (20.98)	120 (41.24)
1 - mild	15 (4.00)	–	65 (29.02)	72 (24.74)
2 - moderate	136 (36.27)	–	81 (36.16)	55 (18.90)
3- severe	221 (58.93)	–	31 (13.84)	44 (15.12)
Hematocrit (%)	35.78 ± 5.76	36.77 ± 5.02*	37.98 ± 4.99*	38.59 ± 4.67*
Hemoglobin (g/dl)	11.72 ± 2.08	12.14 ± 1.87*	12.56 ± 1.82*	12.75 ± 1.66*
Albumin (g/dl)	3.59 ± 0.62	3.78 ± 0.50*	3.85 ± 0.47*	3.99 ± 0.48*
C-reactive protein (mg/dl)	20.22 ± 35.26	9.42 ± 20.9*	7.09 ± 17.12*	5.54 ± 14.29*
Calprotectin (μg/g)	1360.14 ± 1853.06	583.66 ± 843.26*	374.47 ± 521.87*	327.05 ± 589.45*

Data presented as mean ± SD and n (%). *p < 0.05 compared to baseline. *Calculated by Student t test or Pearson, χ2 or Fisher´s exact test

**Fig. 1:**
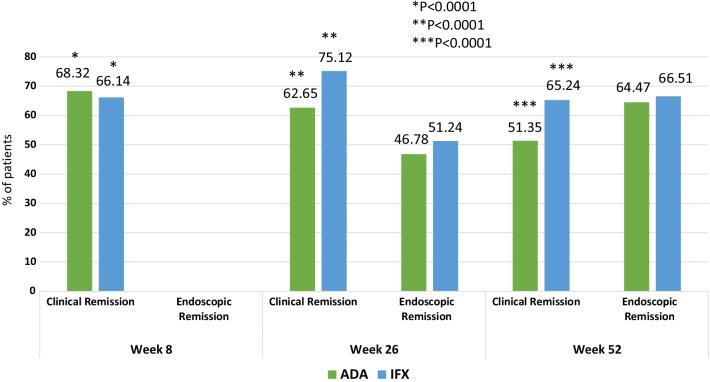
Comparative analysis of clinical and endoscopic remission rates with ADA or IFX in patients with UC at weeks 8, 26 and 52 of treatment

Comparative data is illustrated in Fig. 1. Clinical remission rates were higher in the IFX group at weeks 26 (IFX: 75.12% vs. ADA: 62.65%, *p* < 0.0001) and 52 (IFX: 65.24% vs. ADA: 51.35%, *p* < 0.0001). There was no significant difference in endoscopic remission rates between the groups after 26 and 52 weeks. Table 3 describes in detail a comparative analysis in efficacy, clinical and biochemical parameters between the groups. After induction, clinical remission rate was higher in the ADA group at week 8 (IFX: 66.14% vs. ADA: 68.32%, *p* < 0.0001). C-reactive protein values were lower in the IFX group at weeks 26 (*p* = 0.0181) and 52 (*p* = 0.0008) and fecal calprotectin levels were also lower in the IFX group at week 52 (*p* = 0.0047).

**Table 3:** Comparative analysis between clinical and endoscopic remission between the Adalimumab and Infliximab groups at baseline and at weeks 8, 26 and 52 of treatment

	Baseline			Week 8			Week 26			Week 52		
	Infliximab (n=271)	P-value	Adalimumab (n=101)	Infliximab (n=251)	*P* value	Adalimumab (n=83)	Infliximab (n=213)	*P* value	Adalimumab (n=76)	Infliximab (n=215)	P-value Adalimumab (n=103)	
Mayo partial score	5.42 ± 2.50	5.88 ± 2.25	0.0885	3.16 ± 2.56	3.45 ± 2.29	0.1831	2.72 ± 2.51	2.35 ± 2.15	0.0455	2.25 ± 2.58	1.75 ± 2.10	0.0049
Mayo endoscopic score	2.50 ± 0.70	2.54 ± 0.58	0.8312	-	-	-	1.50 ± 0.92	1.40 ± 0.99	0.5801	1.22 ± 1.14	1.03 ± 1.08	0.1584
Mayo total score	8.06 ± 2.64	8.49 ± 2.41	0.1929	-	-	-	4.34 ± 2.72	3.72 ± 2.77	0.0162	3.46 ± 3.21	2.68 ± 2.71	0.0006
Clinical remission	20 (19.42)	34 (12.55)	0.057	69 (68.32)	166 (66.14)	<0.0001	52 (62.65)	160 (75.12)	<0.0001	57 (51.35)	184 (65.24)	<0.0001
Clinical response	-	-	-	56 (56.00)	159 (63.35)	0.2022	66 (82.50)	178 (84.36)	0.7002	62 (84.93)	182 (88.35)	0.4487
*Mayo endoscopic subscore*												
0 - remission	3 (2.91)	-					10 (16.13)	37 (22.84)		26 (34.21)	94 (43.72)	
1 - mild	7 (6.79)	8 (2.94)					19 (30.65)	46 (28.40)		23 (30.26)	49 (22.79)	0.114
2 - moderate	27(26.20)	109 (40.07)	0.002	-	-	-	25 (40.32)	56 (34.57)	0.681	11 (14.47)	44 (20.79)	
3 - severe	66(64.08)	155 (56.98)					8 (12.90)	23 (14.20)		16 (21.05)	28 (13.02)	
Hematocrit (%)	36.18 ± 5.60	35.62 ± 5.82	0.4225	36.47 ± 5.44	36.88 ± 4.87	0.5440	37.54 ± 5.59	38.15 ± 4.72	0.3670	38.98 ± 5.11	38.45 ± 4.52	0.4435
Hemoglobin (g/dl)	11.95 ± 2.01	11.64 ± 2.10	0.2082	12.10 ± 2.08	12.16 ± 1.79	0.8106	12.29 ± 2.04	12.67 ± 1.72	0.1219	12.81 ± 1.82	12.73 ± 1.60	0.7655
Albumin (g/dl)	3.59 ± 0.66	3.59 ± 0.60	0.9821	3.84 ± 0.56	3.76 ± 0.47	0.3826	3.79 ± 0.44	3.88 ± 0.48	0.2484	3.88 ± 0.50	4.03 ± 0.47	0.0885
C-reactive protein (mg/dl)	19.94 ± 37.99	20.33 ± 34.29	0.8725	10.49 ± 19.55	8.98 ± 21.46	0.3807	9.25 ± 20.33	6.22 ± 15.63	0.0181	8.59 ± 15.68	4.50 ± 13.68	0.0008
Calprotectin (μg/g)	1084.27 ± 929.60	1500.49 ± 2172.67	0.1790	520.60 ±447.67	616.84 ±995.01	0.5533	320.77 ±406.86	405.50 ±580.11	0.4439	489.02 ±777.18	178.21 ±272.71	0.0047

Data presented as mean ± SD and n (%). Calculated by Student’s t test or Pearson, χ2 or Fisher’s exact test

Table 4 describes additional efficacy and safety parameters. The mean time of treatment duration was longer with IFX as compared to ADA (41.23 ± 33.14 vs. 28.93 ± 23.36 months, *p* < 0.001). Secondary loss of response rates and need for anti-TNF dose optimization were more frequent in the ADA group (*p* < 0.001). Moreover, more patients in the ADA group needed to switch biological therapy as compared to IFX (*p* = 0.015). Colectomy rates were higher in the IFX group (*p* = 0.007). Adverse events were reported in 13 (11.71%) patients in the ADA group and in 44 (16.67%) patients in the IFX group (*p* < 0.0001). There were no differences between the groups in infectious adverse events. Overall, 4 patients died during anti-TNF treatment (1 in ADA group and 3 in IFX group), because of severe colitis or infection.

**Table 4:** Additional efficacy and safety data compared between the groups

	Adalimumab (ADA) (n=111)	Infliximab (IFX) (n=282)	*P* value
Time of treatment with anti-TNF (months)	28.93 ±23.36	41.23 ±33.14	<0.001
Secondary loss of response	44 (44.00)	96 (36.92)	<0.001
Anti-TNF dose optimization	42 (40.78)	101 (38.55)	<0.001
Switch of biological therapy	37 (35.58)	61 (23.28)	0.015
Colectomy	5 (4.50)	18 (6.59)	0.007
Overall adverse events	13 (11.71)	44 (16.67)	0.3902
Infectious	7 (6.93)	25 (9.51)	0.4373
Infusion or injection reactions	3 (2.97)	6 (2.42)	0.7218
Other adverse events	9 (9.0)	25 (9.54)	0.8744
Death	1 (1.02)	3 (1.17)	0.317

Data presented as mean ± SD and n (%). Patients could have more than one adverse event. Calculated by Student *t* test or Pearson, χ^2^ or Fisher´s exact test

**Fig. 2:**
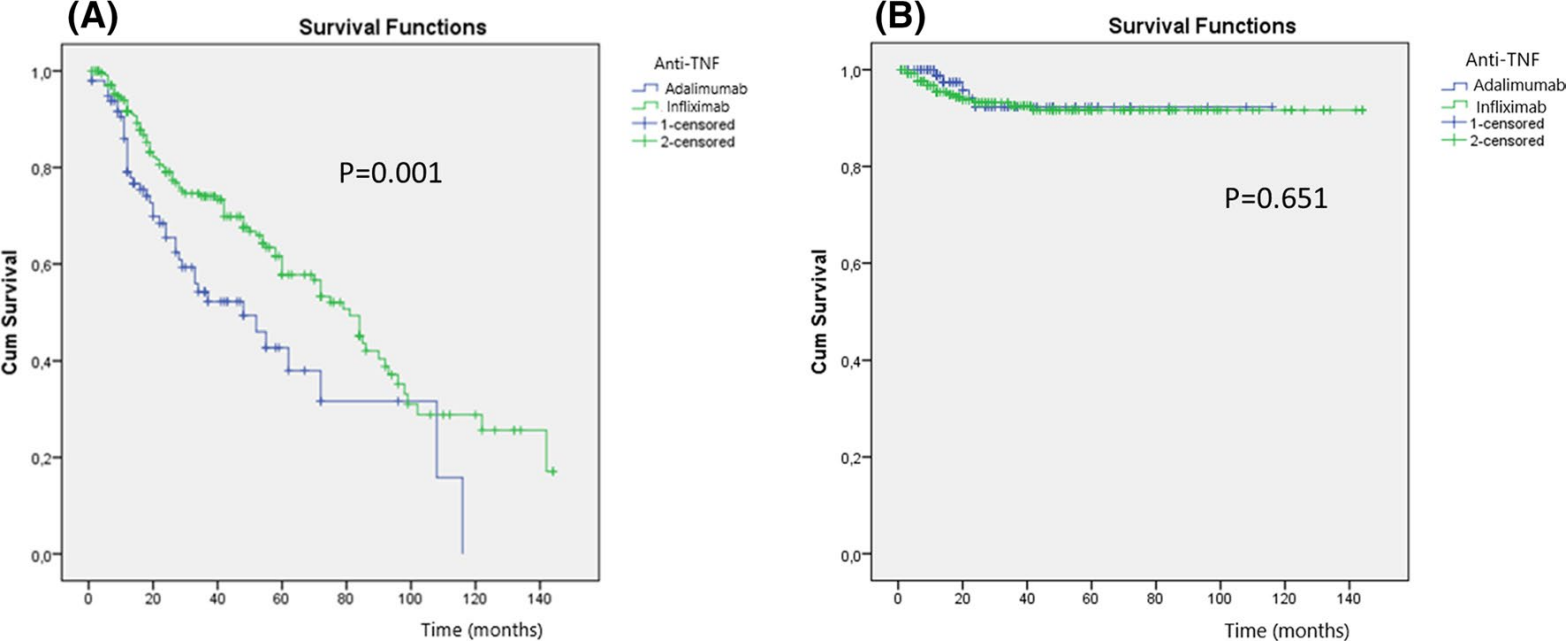
Kaplan–Meier survival curves showing the relationship between loss of response (**A**) and colectomy (**B**) according to anti-TNF therapy. Loss of response was lower in the Infliximab compared to the Adalimumab group (*p* = 0.001). There was no difference regarding colectomy rates between the groups (*p* = 0.651)

According to the Kaplan–Meier survival curve, loss of response was less frequently observed in Infliximab as compared to the Adalimumab group (*p* = 0.001), Fig. 2A. There was no difference regarding colectomy rates between the groups (*p* = 0.651), Fig. 2B.

### Factors associated with clinical and endoscopic remission at week 52

According to logistic regression analysis (Table 5), variables associated with clinical remission at week 52 were age (OR 1.052, 95%CI 1.026–1.080, *p* = 0.0001), total Mayo score at baseline (OR 0.866, 95%CI 0.755–0.994, *p* = 0.0401), no prior exposure to biological therapy (OR 2.903, 95%CI 1.423–5.922, *p* = 0.0034), use of IFX (OR 1.980, 95%CI 1.040–3.759, *p* = 0.0378), clinical response at week 26 (OR 4.778, 95%CI 2.208–10.339, *p* < 0.0001) and endoscopic remission at week 26 (OR 4.909, 95%CI 2.295–10.500, *p* < 0.0001).

**Table 5:** Univariate logistic regression model with associated factors for clinical and endoscopic remission at week 52 of treatment in UC patients

	Clinical remission			Endoscopic remission		
	Odds Ratio	95% Confidence interval	*P* value	Odds Ratio	95% Confidence interval	*P* value
Age (y)	1.052	1.026–1.080	0.0001	1.008	0.990–1.026	0.3998
Gender (female vs. male)	1.481	0.801–2.739	0.2105	1.493	0.910–2.449	0.1129
BMI (kg/m^2^)	1.027	0.958–1.101	0.4596	1.006	0.955–1.060	0.8291
Active smoking (yes x no)	1.741	0.503–6.021	0.3813	1.537	0.627–3.770	0.3478
Time between diagnosis and onset of anti-TNF (y)	1.035	0.970–1.105	0.2954	0.987	0.942–1.035	0.6015
Presence of EIM	1.110	0.579–2.129	0.7538	1.195	0.711–2.007	0.5011
Corticosteroid at baseline	0.509	0.235–1.102	0.0868	0.648	0.368–1.140	0.1320
Azathioprine use	1.105	0.528–2.314	0.7908	1.158	0.641–2.093	0.6274
Total Mayo score at baseline (points)	0.866	0.755–0.994	0.0401	1.014	0.917–1.121	0.7879
No previous use of biological therapy	2.903	1.423–5.922	0.0034	2.000	1.056–3.787	0.0333
Anti-TNF therapy (IFX vs. ADA)	1.980	1.040–3.759	0.0378	1.0941	0.6325–1.8939	0.7472
Clinical response at week 8	1.848	0.986–3.465	0.0555	1.770	1.059–2.957	0.0293
Clinical response at week 26	4.778	2.208–10.339	< 0.0001	7.341	3.228–16.695	< 0.0001
Endoscopic remission at week 26	4.909	2.295–10.500	< 0.0001	8.280	4.138–16.571	< 0.0001
Loss of response (No vs. Yes)	5.042	2.506–10.146	< 0.0001	6.489	3.701–11.379	< 0.0001
Drug optimization (No vs. Yes)	4.913	2.458–9.819	< 0.0001	6.355	3.651–11.064	< 0.0001

*EIM* extraintestinal manifestations. *BMI* body mass index

Variables associated with endoscopic remission at week 52 were no prior exposure to biological therapy (OR 2.0, 95%CI 1.056–3.787, *p* = 0.0333), clinical response at week 8 (OR 1.770, 95%CI 1.059–2.957, *p* = 0.0293), clinical response at week 26 (OR 7.341, 95%CI 3.228–16.695, *p* < 0.0001) and endoscopic remission at week 26 (OR 8.280, 95%CI 4.138–16.571, *p* < 0.0001). The type of anti-TNF used was not associated with endoscopic remission at week 52.

**Fig. 3:**
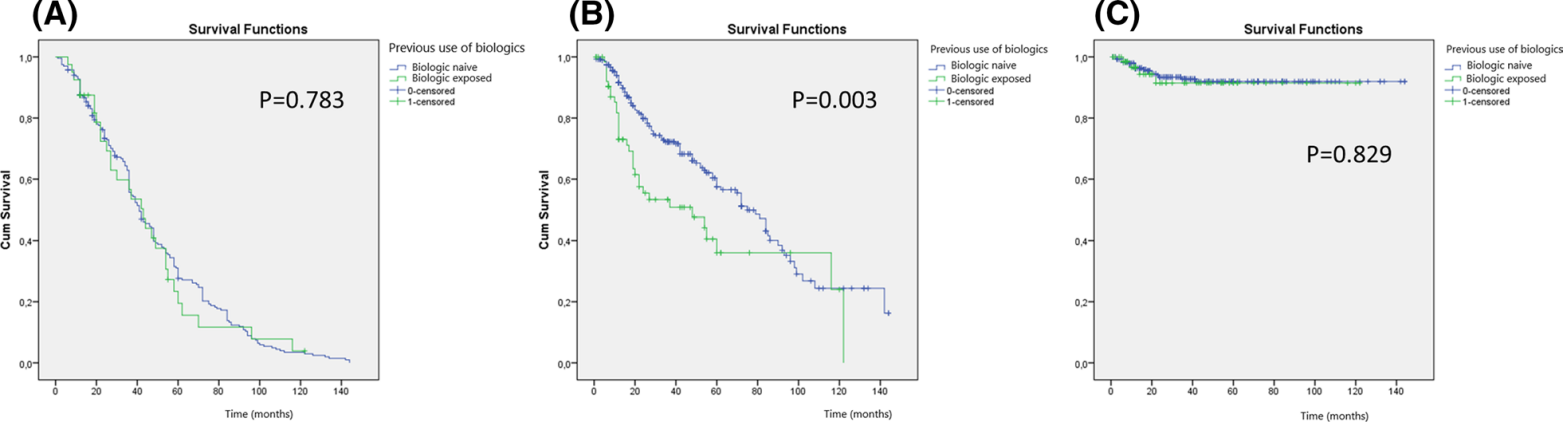
Kaplan–Meier survival curves showing the relationship between clinical remission at week 52 (**A**), loss of response (**B**), and colectomy (**C**) according to previous exposure to biologic therapy. Biologic naïve patients showed lower probability of loss of response as compared to biologic exposed patients (p = 0.003)

According to the Kaplan–Meier survival curve, clinical remission at week 52 was not different between biologic naïve or biologic exposed patients (*p* = 0.783), Fig. 3A. On the other hand, biologic naïve patients showed a lower probability of loss of response as compared to biologic exposed patients (*p* = 0.003), Fig. 3B. Colectomy rates were not different between the groups, Fig. 3C.

## Discussion

This study reports the indirect retrospective comparison between IFX and ADA in a multicenter Brazilian cohort of UC patients. This population of predominantly biological-naive patients (80.6%) showed an overall clinical response rate at week 8 of 61.25%, and the proportion of patients in clinical remission at weeks 8, 26 and 52 was 66.78%, 71.62 and 82.82%, respectively. Clinical remission rates were significantly higher in patients treated with IFX at weeks 26 and 52. Overall, endoscopic remission was observed in 65.98% at week 52, with no differences between the two groups. Older age, no prior exposure to biological therapy, treatment with IFX, clinical response at week 26 and endoscopic remission at week 26 were associated with higher rates of clinical remission at week 52.

Since the approval of IFX [5], the first biological agent for the management of UC, the landscape of medical treatment of the disease has drastically improved, especially for individuals with moderate to severe disease and those refractory to conventional therapies. Biological therapy, mainly TNF-alpha inhibitors, has been associated with clinical remission, endoscopic healing, reduction in the need for hospitalizations and colectomy [16]. Data supported by randomized controlled trials have demonstrated that both IFX and ADA are effective in inducing and maintaining clinical remission in patients with moderate to severely active UC [5, 6], although clinical remission rates after 1 year were not higher than 20.5% for both agents.

The results of the present study are in tune with the reported efficacy of these agents in the real-world scenario, where the proportion of responders after 52 weeks of exposure varies from 30 to 65% for ADA [7–10, 13, 17, 18] and from 39 to 70% for IFX [11, 19–22], respectively. The discrepancy in the performance of biologics between the real-world scenario and pivotal studies is usually attributable to the restriction of concomitant treatments that could favor a response and the required washout period between one drug and another in the design of randomized clinical trials. However, it is important to emphasize that there is significant heterogeneity in methodology, patient populations and clinical scoring systems among the available real-world studies in UC, which limits extensive comparisons with our results.

In the present study, there was a statistically significant difference in the proportions of remitters in the IFX group vs. the ADA group favoring ADA at week 8 and IFX in both weeks 26 and 52. The higher efficacy with ADA after 8 weeks in our study is probably a consequence of unadjusted confounding factors indicating more severe disease at baseline in patients treated with IFX, what could have reduced efficacy numbers in the IFX group. In a recent systematic review and network meta-analysis of important trials in biologic-naïve UC patients, IFX, ADA and vedolizumab were superior to placebo, and vedolizumab was superior to ADA for maintenance of clinical remission and endoscopic improvement in patients who responded to induction therapy [23]. Several real-life studies comparing the efficacy of different anti-TNF agents demonstrated controversial findings. A recent observational retrospective Italian study comparing IFX, ADA and golimumab observed a better treatment effectiveness in patients treated with IFX as compared to other treatments with the lowest percentages of response rates in all outcomes in patients treated with golimumab (*p* < 0.01). However, after applying a propensity analysis, no statistically difference in each outcome evaluated was identified [8]. Accordingly, a recent Korean retrospective study reported no significant differences between IFX and ADA treatment in the rate of clinical remission or clinical response at 8 or 52 weeks [11]. Conversely, the U.S. cohort study using a large administrative claims database showed that the risk of corticosteroid use was significantly lower in IFX-treated patients, as compared to ADA (HR, 0.82; 95% CI, 0.68–0.99), although the risk of hospitalization and serious infections were comparable [24]. These results from different countries demonstrate the variation in findings in the real-world setting.

Despite advances in medical management of UC with the introduction of biologics and small molecules, the potential for disease modification in terms of colectomy rates in the biological era remains unclear. Our study showed a colectomy rate of 4.5% in patients who used ADA and 6.59% with IFX, in a follow-up of 28.93 ± 23.36 and 41.23 ± 33.14 months, respectively. This number can be compared to other similar studies. A recent Swiss population-based study assessing colectomy rates in UC patients demonstrated a significantly decrease in colectomy rates for UC over time after 2005 [25]. Accordingly, a Canadian population-based study showed a significant decrease in the temporal trends of elective colectomy rates from 1997 to 2009, along with a marked increase in the prescriptions of infliximab after 2005 [26]. Although this time-trend decrease might also be related to improvements in care, including earlier diagnosis and adoption of guideline recommendations in clinical practice, the decreasing trend suggests a potential role of biologics as disease-modifying agents in the natural course of UC. It may take time to observe this trend in cohort studies around the globe. More population-based data from Brazil regarding reduction of colectomy rates over time in UC are awaited.

In the present study, prior exposure to another biologic was associated with a lower chance of long-term clinical remission, which may reflect disease severity at baseline. This is a common practice in real setting, what is captured in observational studies as ours. Data evaluating the influence of previous exposure to biologics in response to anti-TNF treatment is conflicting. The randomized, double-blind, placebo-controlled ULTRA 2 trial demonstrated that among patients who had previously received anti-TNF agents, rates of remission at week 8 were 9.2% on ADA patients and 6.9% on placebo (*p* = 0.559); corresponding values for week 52 were 10.2% and 3% (*p* = 0.039) [6]. Conversely, in a retrospective cohort study from the Spanish ENEIDA registry, response to prior treatment with IFX was the only predictive factor of response to ADA at week 12, which was observed in 90% of IFX remitters, 53.8% of responders and 33.3% of primary non-responders (*p* = 0.01). These observations should be interpreted with caution given that most of studies do not assess whether previous treatment was discontinued due to pharmacokinetic failure, immunogenicity, or mechanistic failure.

Early response to anti-TNF treatment has consistently been reported as a predictive factor of higher long-term remission rates. Data from an Italian cohort of ADA-treated patients showed that clinical remission and low C-reactive protein at week 12 predicted clinical remission at week 54 (OR 4.17, 95% CI 2.36–19.44; OR 2.63, 95% CI 2.32–14.94, respectively). Likewise, a Swedish retrospective multi-center study of IFX treatment in UC patients identified non-response at 3 months as an independent risk factor for poor outcome, predicting subsequent colectomy [22]. We observed similar results in the present study since clinical response at week 8 was associated with endoscopic remission and clinical response at week 26 was associated either with clinical and endoscopic remission at week 52.

Data derived from two meta-analysis and a pooled analysis of IBD trials have demonstrated no increased risk of serious infections in antitumor necrosis factors-treated patients compared with placebo [27–29]. In our study, anti-TNF treatment was well tolerated, and no new safety signals were observed, with no difference between the groups. Apart from that, the percentage of adverse events in each group was low in comparison to pivotal trials and other retrospective studies. Comparative safety analyses between different anti-TNFs are limited in the literature. Through the analysis of a health insurance database, it was demonstrated that subcutaneously administered anti-TNFs exhibited a higher risk of serious infections (HR, 1.34; 95% CI, 1.18–1.53) than intravenous anti-TNF [30]. On the other hand, a retrospective study in pediatric IBD patients observed a higher overall incidence of infections in infliximab compared with adalimumab-treated patients [31]. Additionally, a Brazilian retrospective single-center study demonstrated no differences between ADA and IFX patients in CD management (63.2% with IFX and 64.5% with ADA, *p* = 0.879), with no differences in infections or treatment interruption [32]. The lower numbers of adverse events in our UC multicentric national study are probably associated to limitations in data capturing by different physicians. The infusion site reaction rate was in line with previous reports. Data from TREAT registry reported an incidence of 2.8% with IFX in terms of infusion reactions, most common being headache and arthritis [33] while injection-site reactions were reported in an incidence of 0.1/100 patient-years in the adalimumab safety PYRAMID registry [34]. These numbers were comparable to the findings of our study.

Our study is associated with some limitations which need to be considered before the final analysis of the results. Firstly, the sample size was limited considering the increasing incidence and high estimated prevalence of UC in Brazil (estimated prevalence of 66.45 per 100,000 in 2020) [35]. However, this was unavoidable considering that biologics were just recently reimbursed for UC management in Brazil and that penetration of anti-TNF agents in Latin America is lower in UC as comparable to the rest of the world [36]. Another important limitation was a natural selection bias, a common feature of the retrospective and observational study design, where more severe patients could be directed to IFX use. However, these findings highlight the that physicians may confront in the management of IBD patients in our country [37]. The observational nature of this study carries the inherent biases associated with the retrospective study design. In addition, not all information regarding clinical scores were readily available in all timepoints of interest in medical charts, which limited the assessment of treatment response. No patients with golimumab were included, demonstrating the lack of experience with this agent in our country. Safety analysis was probably underestimated, due to bias in data collection. Another important point is that a systematic evaluation of Mayo endoscopic subscores was not available for the entire population of patients. Lastly, not all patients included in the study presented moderate-to-severe activity at induction, which may have interfered with the final results. As this is the first multicenter study in the country with the aiming to evaluate the use of anti-TNF in patients with UC, we found it interesting to evaluate the scenario and epidemiological profile of all patients with indication for the use of the medication and, therefore, the study inclusion criteria were more comprehensive. We are aware that other study designs would be more appropriate for the study, such as propensity score matching, or the inclusion of biologic naïve patients, exclusively. Further studies can clarify these issues in the future. Despite these limitations, our study provides insightful information as being the first multi-center Brazilian study to report on long-term outcomes of anti-TNF treatment in UC patients, from both private and public settings.

## Conclusions

In summary, in this national Brazilian retrospective study, anti-TNF therapy with IFX and ADA were effective in the management of UC. Clinical remission rates were higher with ADA at week 8. On the other hand, IFX was associated with higher rates of clinical remission at weeks 26 and 52 in comparison to ADA. Patients naive to biological therapy presented higher rates of clinical and endoscopic remission.

## Data Availability

The datasets used and/or analysed during the current study are available from the corresponding author on reasonable request.

## Abbreviations

ADA: Adalimumab
BMI: Body Mass Index
CD: Crohn’s disease
CI: Confidence interval
EIM: Extraintestinal manifestations
IBD: Inflammatory bowel disease
IFX: Infliximab
OR: Odds Ratio
TNF: Tumor necrosis factor
UC: Ulcerative colitis
